# Intercultural discussion of conceptual universals in discourse: joint online methodology to bring about social change through novel conceptualizations of Covid-19

**DOI:** 10.1057/s41599-022-01230-4

**Published:** 2022-06-25

**Authors:** Zsuzsanna Schnell, Francesca Ervas

**Affiliations:** 1 University of Pécs, Pécs, Hungary; 2 University of Cagliari, Sardinia, Italy

**Keywords:** Language and linguistics, Language and linguistics

## Abstract

The present article addresses the professional conclusions of an international platform of education in intercultural discourse in the European Union’s EDUC Project. In flagging social issues and concerns, *cross-cultural academic collaboration* is a powerful tool to bring about *social change*. In our educational project participants encounter different cultures, so the discussed topics, and especially the metaphors for the Covid-19 pandemic, receive instant reflections from *different cultural perspectives*, multiplying the potential sphere of valid *interpretations*, yielding novel perspectives in *intercultural pragmatics* and communication. This gives birth to a *novel methodology* that builds on the open-minded integration of different points of view, understanding universal traits of human cognition and differences in culture in the linguistics of discourse.

## Background

The purpose of the present study is twofold. Firstly, we aim to describe a novel platform for *academic discourse*, namely, a joint course based online methodology stemming from the *collaboration of scholars*, highlighting its efficiency in *discursive strategies targeting value judgments* with the desire to bring about *social change*.

Secondly, we aim to explore the *socio-cultural and linguistic dimensions* of the conceptualization of Covid-19 and related issues of the pandemic, highlighting the power of *symbolic and material aspects of discourse* through the investigation of the *conceptual framework* Covid-19 metaphors provide to help us overcome the difficulties of the pandemic. Through *cross-cultural academic discourse we identify* the *processes* that *bring about social change* by highlighting the unique *methodology* that makes it possible to understand how culture drives behavior and how our cultural conceptual frameworks given by conceptual metaphors drive our conceptualization (Lakoff and Johnson, 1980; Schnell et al., 2019; Schnell et al., 2021; Ervas et al., 2017) and how this can be altered by *academic discourse* emphasizing *objectivity*, *impartiality, and consistency of evaluation*.

### EDUC—The European Union’s online educational collaboration platform for social change

EDUC is an online educational platform, a collaboration for EU countries to provide a professional framework for education at an international level (https://educalliance.eu/). Among the six participating Universities the University of Cagliari and Pécs launched its joint course with the potential of opening its doors to global citizens of the intercultural EDUC framework.

**Fig. 1:**
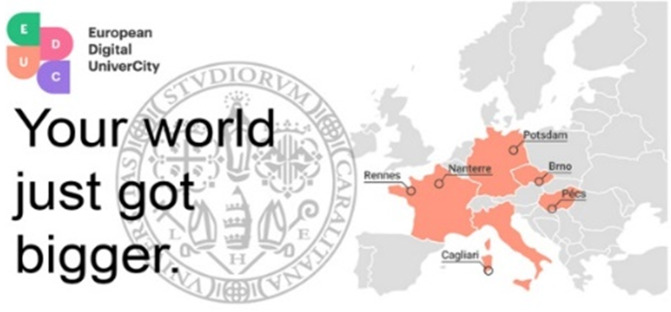
EDUC alliance of academic scholars for novel methodology in online collaborative education. For further sources on the EDUC platform and the course see References.

By joining this collaboration of academic scholars, each university connects its students with several foreign universities and eventually with the entire globalized intercultural world of education. EDUC develops a shared internationalization strategy in accordance with the Sustainable Development Goals (SDGs) to open its community and its services to the world. This novel platform develops a joint research agenda to *enhance a multidisciplinary approach* to tackle global challenges, *speed up the dissemination and transfer of knowledge* in society to unite forces to bring about social change (Fig. 1).

### Introduction to the course and its methodology

The novel approach in the online collaborative framework offers exceptional possibilities for participants to experience cultural immersion in their own classroom, and to discuss the relevant questions at hand from very different perspectives. Due to the online joint methodology, the multicultural reflections on the topic at hand are instantaneous, thus facilitating the integration of perspectives, which gradually and naturally makes participants more open-minded by the end of the course. It enables a type of academic discourse where participants from all cultures and in different parts of the world can discuss questions on the spot from multiple perspectives, enhancing the power of discourse in accepting cultural, social-cognitive and linguistic differences and in overcoming societal challenges by a change in *conceptualization* through *metaphors*, in order to change behavior (Lakoff-Johnson, 1980; Schnell, 2007, 2019; Schnell et al., 2019; Ervas, 2017).

### The course: Collaborative academic discourse with intercultural online methodology

**Fig. 2:**
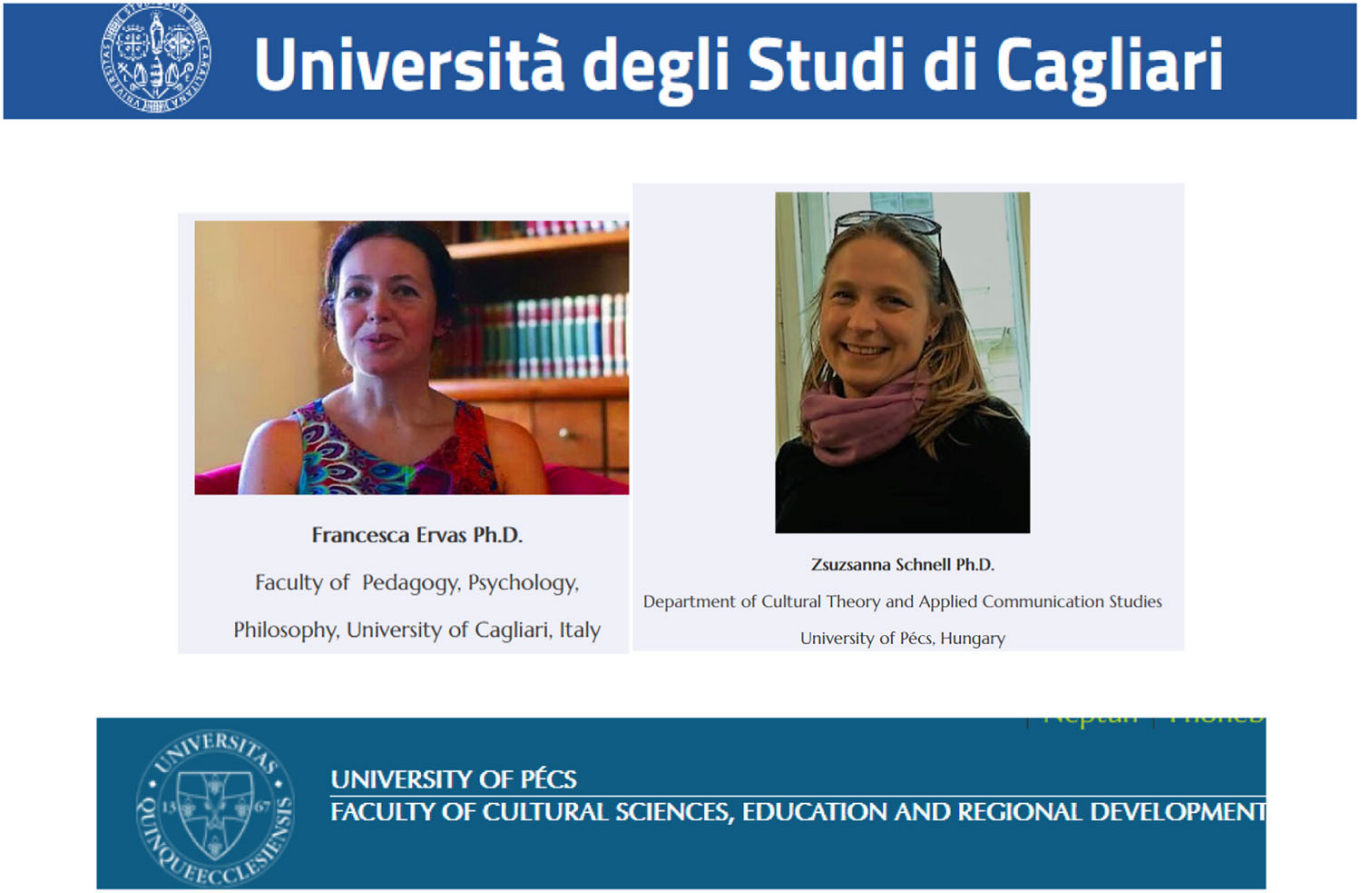
Participating institutions and scholars in the EDUC course devoted for a novel online joint course methodology for a new type of academic discourse.

The EDUC joint course is a result of a long-standing collaboration between two researchers in the fields of philosophy of language and pragmatics (Fig. 2), with the aim to cover issues in metacognition, developmental aspects of cognitive psychology and cognitive linguistics, and intercultural pragmatic issues of conceptual metaphor, irony and communication.

**Fig. 3:**
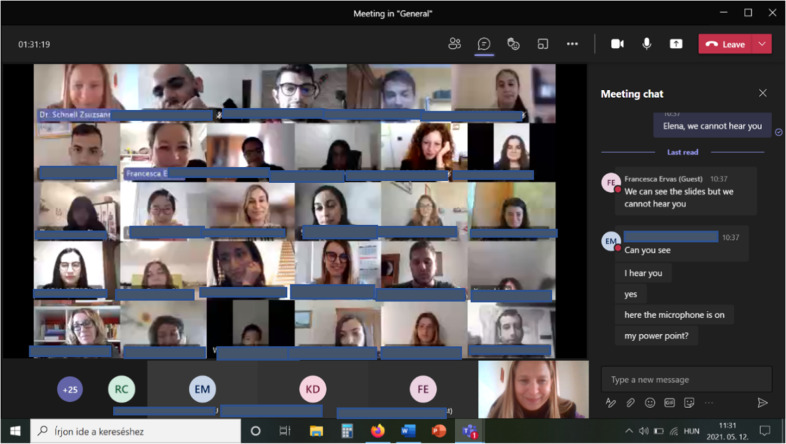
EDUC course participants in online collaboration.

Figure 3 shows the participants in the process of the online joint course framework. (For more information on students’ reflections on the course, its outcome and methodology, see References Section Student’s reflections URL link).

#### Course methodology: efficient knowledge transfer in cross cultural academic discourse

The course methodology focused on the uncovering of culture-specific and universal conceptualization of discourse, by discussing conceptual metaphors to reveal manipulation in fake news in political discourse, and investigating different frames of interpretation offered by conceptual metaphors to bring about social change. The EDUC course connected students from several continents, cultures and countries, ranging from Western individualist cultures (represented by Italy and the Netherlands) through Central-European perspectives (Hungary) with different social- and historical traditions, to collectivistic cultures at the other end of the cultural spectrum, represented by Middle-East (Turkey) and Far-East cultures (China). It was especially interesting to discuss Chinese perspectives, as their role in Covid-19 management was crucial, and to see the social and emotional effects in their conceptualization and use of discursive strategies targeting the saving of Face/Social Self and the culture-specific aspects of the local management of discourse (Goffman, 1955).

#### Course objectives and outcomes: identifying processes that bring about social change

The unique multicultural aspect enhanced the possibilities of intercultural discourse in understanding universals and culture-specific traits of human conceptualization. The timely issue of Covid-19 also presented new possibilities in the investigation of health metaphors and the conceptualization of Covid-19 through the metaphors we find in political discourse, which was confronted with a variety of conceptual metaphors from not directly politically driven contexts that opened up new perspectives and revealed new ways of seeing the pandemic itself, as less of a “war”, more of a process of enlightenment, and “possibility for change”. This raised new interpretations and thus new solutions for social concerns centering around Covid-19 pandemic and related issues.

The on-line discussion of several questions about human culture, conceptualization and communication from several cultural perspectives enabled participants to see how the functioning of the human mind is largely determined by conceptual metaphors (Kövecses, 2005), showed how universal these might be, and revealed some cultural variations in the conceptualization of the pandemic that may have the power to trigger social change (Schnell, 2014, Schnell et al. 2021, Ervas, 2017). This brought about a significant transfer of knowledge and a change in conceptualization crucial for long-term changes in the behavior and functioning of human societies.

## Conceptualization, culture, metaphor

**Fig. 4:**
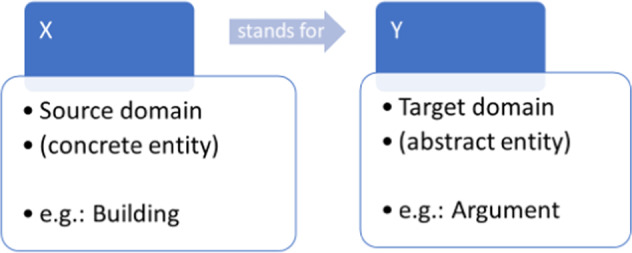
Conceptual metaphor functioning as a cognitive model.

Since the influential works by Lakoff and Johnson (1980, 1999) on the conceptual nature of metaphors, it is almost universally accepted in cognitive linguistics and psycholinguistics that the role of metaphor in human language and cognition is essential, as they are not merely linguistic entities, but in fact, constitute a fundamental feature of the human mind. Metaphors thus act as cognitive models in our thinking and in the functioning of the human mind, as they provide a model for a concept. Metaphors are also defined as tool for the understanding of one thing in terms of another: an abstract (difficult, intangible) concept in terms of something physically tangible, easy to imagine and often visible, known to use and is therefore used as a model for the transfer of knowledge to conceptualize the abstract, difficult concept (mostly emotions, feelings, relationships, interpersonal or psychological, mental processes or complex phenomena like a pandemic, social equality, etc.). Figure 4. shows the conceptual nature of metaphors, based on two domains (*X*: source domain (concrete entity) and *Y*: target domain (abstract entity) in understanding one thing in terms of another (*X* = *Y*) (Kövecses, 2002).

**Fig. 5:**
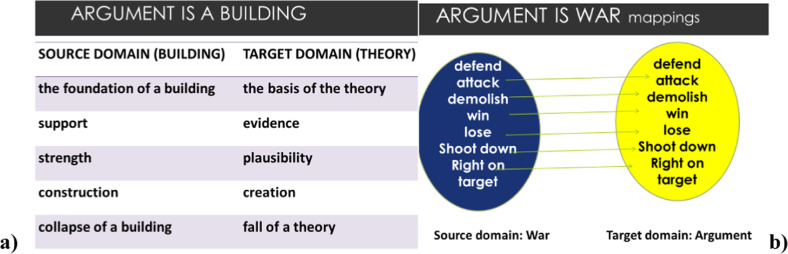
**a**, **b** Correspondences in conceptual metaphor between the domains, shaping our conceptualization.

These models eventually shape and determine our concepts and conceptualization of abstract entities in the world, as they influence our understanding and perspective on the given topic. For example, the abstract notion of Argument (quarrel) is often conceptualized in terms of building (ARGUMENT IS A BUILDING) or as war (ARGUMENT IS WAR conceptual metaphor) by most cultures and humans. Figure 5a, b outlines the *correspondences* between the two domains.

**Fig. 6:**
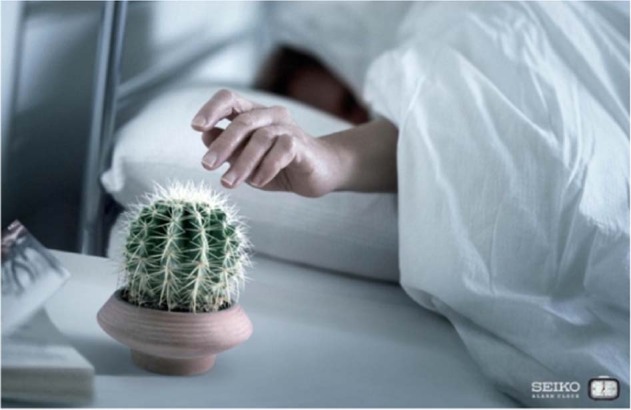
Example of visual metaphor (Credits: SEIKO, Tempitalia; Creative agency: BBDO).

As metaphor is primarily a matter of (embodied) thought (Lakoff and Johnsons, 1980), it can be *represented not only in verbal mode, but also in visual mode* (Forceville, 2008). Thus, the target domain can be depicted or contextually suggested by depicting the source domain, as in Fig. 6, where the creative metaphor of the alarm clock as a cactus is grasped via both the contextual knowledge and action (touching the alarm clock/cactus).

In metaphor processing some features of the cactus (e.g. being spiny, thorny and stinging) are thus projected to the sound of the alarm clock, as something that make us wake up. As for verbal metaphors, we can find visual and multimodal metaphors in a variety of discourse genres, ranging from social campaigns to political posters, to conceptualize the issues at hand (Ervas, 2021; Szécsi, 2013).

Conceptual metaphor theory (CMT) (Lakoff and Johnsons, 1980) has been so influential that it has been prevalent since the late nineties, however, that does not mean it has not been challenged (for an overview see Schnell, 2007, 2015; Ervas et al., 2017). The full description of the criticism of conceptual metaphor theory is, however, beyond the scope of the present article. We investigate the cognitive model function of metaphorical conceptualizations in the Covid-19 pandemic and analyze the effect of the frameworks these metaphors provide on our thinking, conceptualization, culture and behavior. We highlight the powerful effects of using conceptual metaphors in any type of discourse in general and investigate the use of Covid-19 metaphors in political discourse in particular, and we finally suggest a novel framework for conceptualization and thus a novel metaphor for Covid-19 in order to achieve social change. This new conceptual metaphor and its new framework can serve as an alternative and eventually change our understanding of the pandemic and see it as a new possibility for change.

### The cognitive principle in metaphorical conceptualization

**Fig. 7:**
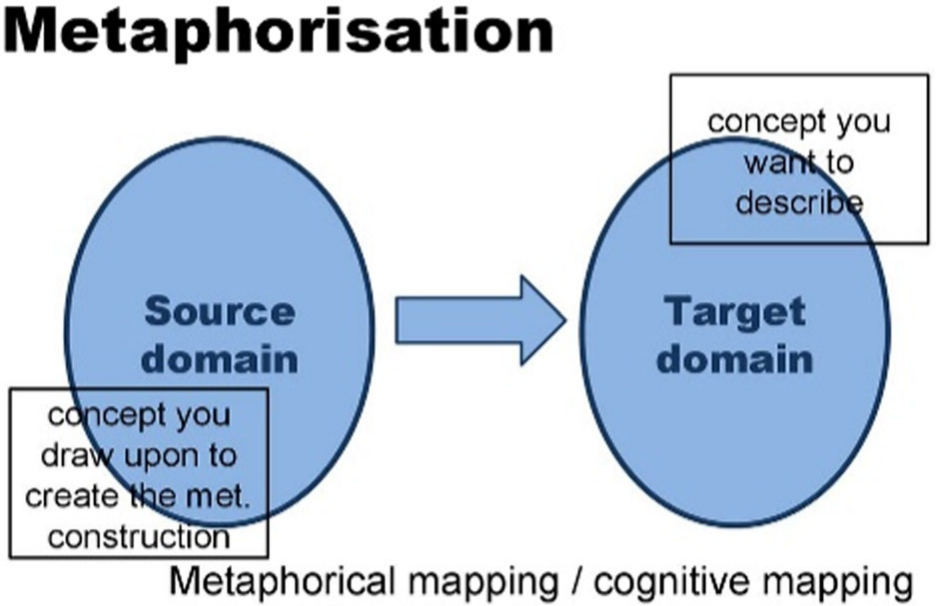
Highlighted properties (“you want to describe”) are the relevant conceptual features.

In metaphor understanding, some features of the source domain are selected to better understand the target domain. However, not all features are relevant in the cognitive model, of course, and certain highlighted features are more striking and important in the conceptualization than others. A critique of the CMT by Fauconnier and Turner, for example, uses the example of the metaphor LAWYERS ARE SHARKS to demonstrate the emerging structure within the metaphorical entailments (Fauconnier and Turner, 1998; Wilson and Carston, 2006; Schnell, 2015), with its own properties leading to emergent structures and optimal relevance (Sperber and Wilson, 1986; Giora, 2001), showing that we do not think of lawyers as water creatures having fins but we conceptualize them in terms of their aggressive behavior, the property the two domains and concepts share. In this perspective, the two domains do not fully overlap, as Fig. 7 shows, with their particular meaning properties in squares represented modularly in the image.

### Conceptual metaphors as models for understanding

The powerful effect of conceptual metaphors is that they are not purely linguistic items, but exist in our long-term memory, constituting cognitive models for conceptualization. These internalized cognitive models (ICMs) serve as a framework for understanding and conceptualization, and as we are mostly unaware of this functioning of the human mind, we sometimes cannot fight its effects either. This is very striking in the case of the current crisis in our society posed by the Covid-19 pandemic where the covid-19 is war conceptual metaphor was widespread in political discourse, determining people’s views about it and the action they were required to take. An understanding of the conceptual nature of the use of metaphors enables us to change the discourse about it; knowledge transfer can help us explore the social, linguistic and cognitive dimensions, and thus help us identify the processes with the power to bring about social change.

### WAR metaphor in health communication

In the field of health communication, the *WAR metaphor* has been largely applied in discourse to describe illness and therapy management, especially in oncology (Ervas et al., 2016; Semino et al., 2018), and thus making easier to conceptualize a phenomenon that was difficult to express in literal terms in patients’ lives.

#### WAR metaphor for cancer

As the WAR metaphor is highly conventional and frequent in discourse about cancer, it is easier to understand compared to completely new metaphor. Even doctors used the WAR metaphor to encourage patients, by depicting them as *heroes*, *warriors* or *fighters* who could *defeat* cancer, thus acknowledging the difficulties to face in the therapy management and making them (and/or their families) feel empowered with a sense of control over the disease. However, criticism has been raised against the use of the *WAR metaphor in discourse about cancer*, highlighting the negative entailments of the metaphor (Sontag, 1978): both patients and doctors reported feelings of anger or sadness in perceiving them as *losers* in a war that was not up to them or in their control, and thus they could not *win*. The WAR metaphor made them feel like weak people, who did not *fight enough* to *beat* the disease.

#### WAR metaphor for Covid-19

More recently, the *WAR metaphor* has been applied to the Covid-19 pandemic, thus shifting its use from the individual context of a patient suffering from cancer to *the social context of entire communities threatened by Covid-19*. In such a new (and unexpected) context, *new key features* of the WAR metaphor emerged (Marron et al., 2020): what was familiar in discourse about cancer was no longer familiar when applied in discourse about Covid-19. At the very beginning of the pandemic, the *war* against Covid-19 expressed the urgency and the risks that health workers faced in *fighting* the invisible *enemy* on the *front lines*, the need for masks and social distance as *weapons* against Covid-19. However, the WAR metaphor for Covid-19 was highly criticized in public debates for its negative implications, such as the idea that health workers decided to be heroes while other essential personnel’s work was minimized. Thus, new metaphors have been proposed in a variety of discourse genres (from social campaigns to political cartoons) to challenge the shortcomings of the WAR metaphor for Covid-19 and find alternative and more suitable metaphors to talk about the social crisis engendered by the pandemic (see the website reference #ReframeCovid Initiative for a collection of Covid-19 metaphors).

## COVID AS WAR conceptual metaphor and its effect on societal behavior

**Fig. 8:**
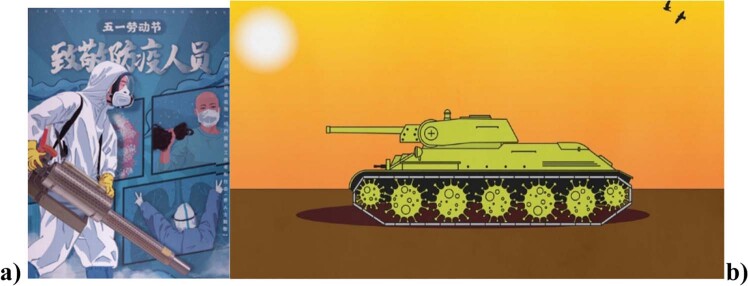
Visual metaphors depicting the conceptualization of Covid as war. **a** Picture published in the online collection “Chinese COVID-19 political propaganda poster collection” of the Princeton University Library, available at the following address: https://catalog.princeton.edu/catalog/99120475213506421; **b** Picture published in the website of the online journal *The Atlantic* at the following address: www.theatlantic.com/international/archive/2020/03/war-metaphor-coronavirus/609049/.

Since Covid-19 is a social crisis, naturally it entails that it has political relevance. To handle the crisis, politics will deal with the pandemic and politicians must deploy efficient strategies to maintain control and avoid anarchy and chaos, and this results in a more centralized, authoritarian communication driven from above, like in hierarchical societies. As a result of this, politicians try to find strategies to tell us what to do, in order to govern the country and lead society out of the crisis. Therefore, politicians will tell us to *fight* the virus, to *kill* the virus, to *win* this *war* against Covid-19. These linguistic expressions clearly show the conceptual metaphor in the background: covid is war, just as many images convey the same idea, functioning as *visual metaphors* (Ervas, 2021), in a variety of discourse genres, ranging from political posters to journal articles, see examples in Fig. 8.

Political discourse communicates that we must fight and win this war against Covid-19, hence, Covid-19 is seen as an intruder, an enemy or an aggressive killer.

### WAR metaphor in political discourse in pandemic times

At the outbreak of the pandemic, in Europe, many politicians used the *WAR metaphor in political discourses*, announcing the measures taken by their country. The French President, Emmanuel Macron, introduced it in his address to the Nation on March 16, 2020, explicitly naming the war and talking about Covid-19 as an invisible enemy that needs (1) a general mobilization and (2) the suspension of the other (normal in peace times) political commitments (for instance, the retirement reform). In his words (from the discourse available at www.elysee.fr/emmanuel-macron/2020/03/16/adresse-aux-francais-covid19):

Nous sommes en guerre, en guerre sanitaire, certes: nous ne luttons ni contre une armée, ni contre une autre Nation. Mais l’ennemi est là, invisible, insaisissable, qui progresse. Et cela requiert notre mobilisation générale. [We are in a war, a sanitary war, we are neither fighting against an army, nor another nation. But the enemy is there, invisible, hard to grasp, and it is spreading. It requires our general mobilisation.]

Nous sommes en guerre. Toute l’action du Gouvernement et du Parlement doit être désormais tournée vers le combat contre l’épidémie. De jour comme de nuit, rien ne doit nous en divertir. C’est pourquoi, j’ai décidé que toutes les réformes en cours seraient suspendues, à commencer par la réforme des retraites. [We are in a war. All action of the government and the parliament must turn towards the combatting of the pandemic. Be it day or night, nothing can distract our attention. This is why I have decided that all the reforms will be suspended, so that we can start reforms to retreat.]

In Italy as well, the Prime Minister Giuseppe Conte associated the health workers’ and law enforcement’s fight against Covid-19 with the Independence war that made Italy united in 1861. At the outset of the second wave of the pandemic, the Hungarian Prime Minister Viktor Orbán said the government was drafting a “war plan” to defend the Nation. The President of the United States, Donald Trump even labelled himself as “a wartime President”, calling the Americans “to make shared sacrifices for the good of the nation” in pandemic times:

To this day, nobody has ever seen like it, what they were able to do during World War II. Now it’s our time. We must sacrifice together, because we are all in this together, and we will come through together. It’s the invisible enemy. That’s always the toughest enemy, the invisible enemy.

### Politicians use motivating language to target our emotions

All participants try to take responsibility in a social crisis, and politicians are no exception. They try to keep their power by getting a grip on the situation, acting fiercely. They also communicate promises in terms of goals and desired future outcomes, and they promise to find a way out of the dangerous situation, calling for unity to *destroy* the virus, as they impose social distancing and lockdown. Social distancing entails keeping defined distance from others, which in extreme cases may lead to physical isolation, and lockdown also isolates individuals in the relative safety of private spaces. Political discourse in the mass media conveys that the new enemy, Covid-19, is attacking us, destroying our habitual life, take everything we had. The reason why such metaphors are used so powerfully in political discourse is that ultimately, their purpose is to enhance cooperation, by frightening people, making them be afraid of the unknown. The press can orient people to distance from each other, as people who are isolated and afraid tend to obey. These metaphors function in a way to unite us, to make us a community, because in a war we are all soldiers, we need to accept this extraordinary situation and thus obey commanders, as we need orders to survive, to avoid chaos. We must fight the enemy, no questions raised, one for all and all for one purpose.

### Metaphor as framing structure in politics

The Conceptual Metaphor Theory argues that our *worldviews* are largely based on *frames* that provide us with *structures* for thinking. Vygotsky, an influential figure of modern cognitive psychology and structuralist views, also emphasized interactionist effects in mental and psychological phenomena, and highlighted the importance of studying structure to understand the genuine notion and phenomenon (Vygotsky, 1978). Metaphors cause the audience to see things, social issues included, in a new light, as metaphors can provide new frames to look at them and new structures to think about them. In *politics*, the explanatory function of metaphors is often subjected to the goal of manipulation, i.e. metaphors are often primarily selected for their emotional and strategic effect. Prime candidates in political speeches in general are metaphorical links with familiar words, e.g. WIND (e.g. *the new breeze*, *the wind of change*), ILLNESS metaphors, e.g.: our country can be healthy; and of course WAR metaphors, e.g. the country has friends, allies and enemies.

## The creativity of metaphor: novel conceptualization by new frames

In the conceptual approach, metaphors are powerful natural cognitive processes that help us understand complex issues in nature and society. They are mediators between the human mind and culture. Novel, non-conventional metaphors (e.g. *Orange is the New Black*) can change both the ordinary language we use and the ways in which we perceive and understand the world. People tend to draw upon experiences in one area of life to give fresh insights and understanding to experiences in another, creating new conceptual realities, often from existing correlations, e.g. LIFE IS A BATTLE./A WOLF IN SHEEP’S CLOTHING/JOHN IS A SHIP WITHOUT A CAPTAIN/LIFE IS WATER IN THE SAND (Schnell et al., 2019).

### Naming, framing and perspective changing functions of metaphors in Covid-19 conceptualizations

Metaphors help us to *give a name to the phenomenon, i.e. the Covid-19 pandemic*, and its consequences in our lives that can otherwise be rather difficult to express in plain language. After all, we resort to metaphors precisely because we experienced a completely unfamiliar and unprecedented collective health phenomenon, which subverted our everyday life. At the same time, the metaphors we use to name the Covid-19 pandemic are never “neutral”, as they also entail a *framing effect* (Entman, 1993, Burgers et al. 2016), i.e. the selection of some relevant features that tacitly determine a particular view and (emotive) evaluation of the target. In the case of Covid-19 pandemic, each metaphor implicitly provides *a specific perspective to interpret what is happening to the social world*. The WAR metaphor not only promote a particular view of society, but also includes specific feelings and attitudes toward the pandemic, which might influence reasoning on the measure to be taken and/or be passively accepted by the society, especially when confirming beliefs already held as true by its members. However, metaphor can also have a *perspective changing function* (Steen, 2008): especially when novel and creative, metaphors can help us in focusing on the target from a completely new perspective, questioning previous beliefs held as true or reframing them, thus providing a new conceptualization that can change our view of the social world.

### Metaphors as powerful tools in discourse: reframing

Effects of reframing are crucial in situations like a social crisis, where we need to find another perspective to discover a new solution. As the famous saying goes, we cannot solve our problems with the same thinking we used when we created them. Finding a new perspective and *new frames for conceptualization* can be essential in overcoming novel societal challenges of the twenty-first century. An example for such a change in conceptualization is exemplified by a novel frame of reference such as a novel Covid-19 metaphor emerging in scientific language. Some articles on Covid-19 explored during the online course actually portray it as a positive event as it awakens us to problems we have in current society. Novel metaphors emerge in scientific language, with new conceptual frames as: covid as enlightenment, covid as collaboration, covid as protector of the ecosystem.

## Joint course methodology in identifying underlying conceptual metaphor and novel frames

In our joint course, the methodology of intercultural pragmatic analysis in a framework of cross-cultural communication was done using MS Teams’ Breakout rooms tool. This allowed the professors to create random rooms across cultures for specific discussions, or create same-culture pairs, groups or rooms for the discussion and exploration of a certain topic. We used both methods to enhance comprehension and involve the students actively in the academic discourse and discussion targeting the discovery of new solutions to bring about social change and tackle challenges in 21st century communities.

In the breakout rooms, for the identification of conceptual metaphors behind the expressions used in political discourse in the press, we collected eight articles with very different perspectives on Covid-19. Some of them portrayed the pandemic as a war, the virus as an enemy, and Covid-19 as an intruder that takes what is ours. Surprisingly, however, some fields, mostly from biology, scientific investigations in natural sciences have a very different perspective, as they see viruses and fully-fledges humans as equal parts of the ecosystem. We gave one article per room for discussion and students had to identify the conceptual metaphor that emerges from the discourse at hand (see the list of articles in the References section).

### Novel conceptualizations in metaphors of Covid-19—an intra-cultural perspective

**Fig. 9:**
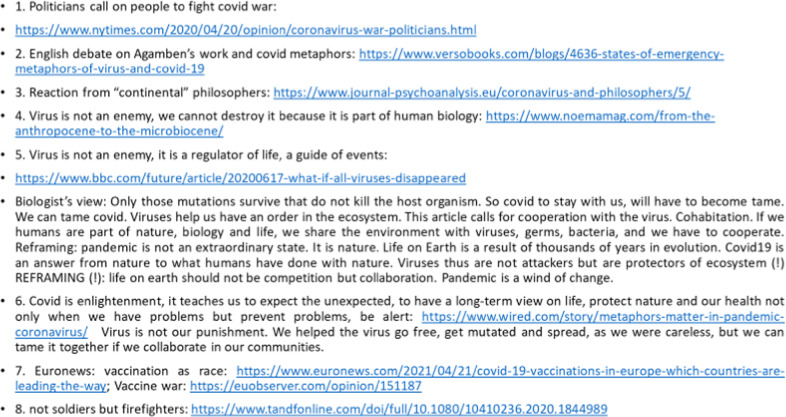
The different conceptualizations of Covid-19 in an intra-cultural spectrum.

The following conceptualizations emerged (Fig. 9), giving us Covid-19 metaphors that ranged from negative (war, killer, intruder) to positive (enlightenment, alarm call, protector of the environment) concepts. This also revealed that cross-cultural conceptualization demonstrates a spectrum of different views, rather than inter-cultural conceptualization, meaning that in politics and in mass media Covid-19 was portrayed as an intruder, and enemy, but in the intra-cultural aspect (within cultures and layers of society, in different disciplinary perspectives) we see differences of conceptualization, for instance, health-care personnel and people are not seen as soldiers but as firefighters (article 8).

Biological views see the virus as a full right player of the ecosystem, just like humans and this basic conceptualization fuels positive aspects of Covid-19, extending this idea to a novel framework which is a possibility for change, therefore protector, savior of living organisms—sharply different conceptualization than the one found in general political discourse. Some sources (article 7) portray vaccination as a race, which itself could be a topic of investigation and is definitely something for future exploration both in terms of metaphoricity, conceptualization and social effects of discourse in and outside the Academia.

## Reframing as a possibility for social change

**Fig. 10:**
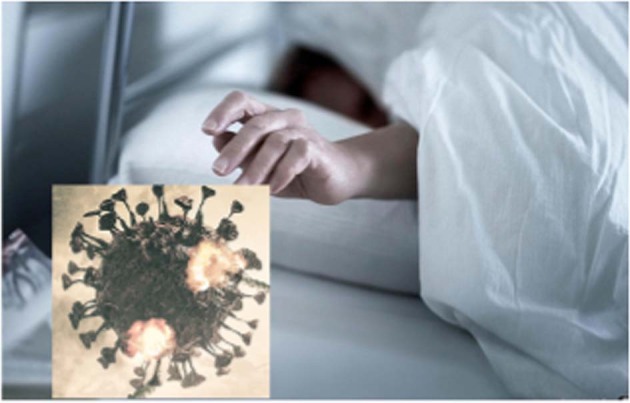
Reframing: COVID AS ALARM CLOCK conceptual metaphor.

In the novel conceptual framework offered by positive metaphors for Covid-19 as covid as enlightenment, covid as an opportunity for change, covid as a savior of the ecosystem, the pandemic is portrayed not as war but as *collaboration*. In this view, Covid-19 stands for enlightenment, a call for change, an alarm clock, telling us it’s time to wake up! Figure 10 shows how metaphors, also in the visual mode, can be reframed (see Fig. 6 for the original source), to bring about a completely new conceptualization of a pandemic.

**Fig. 11:**
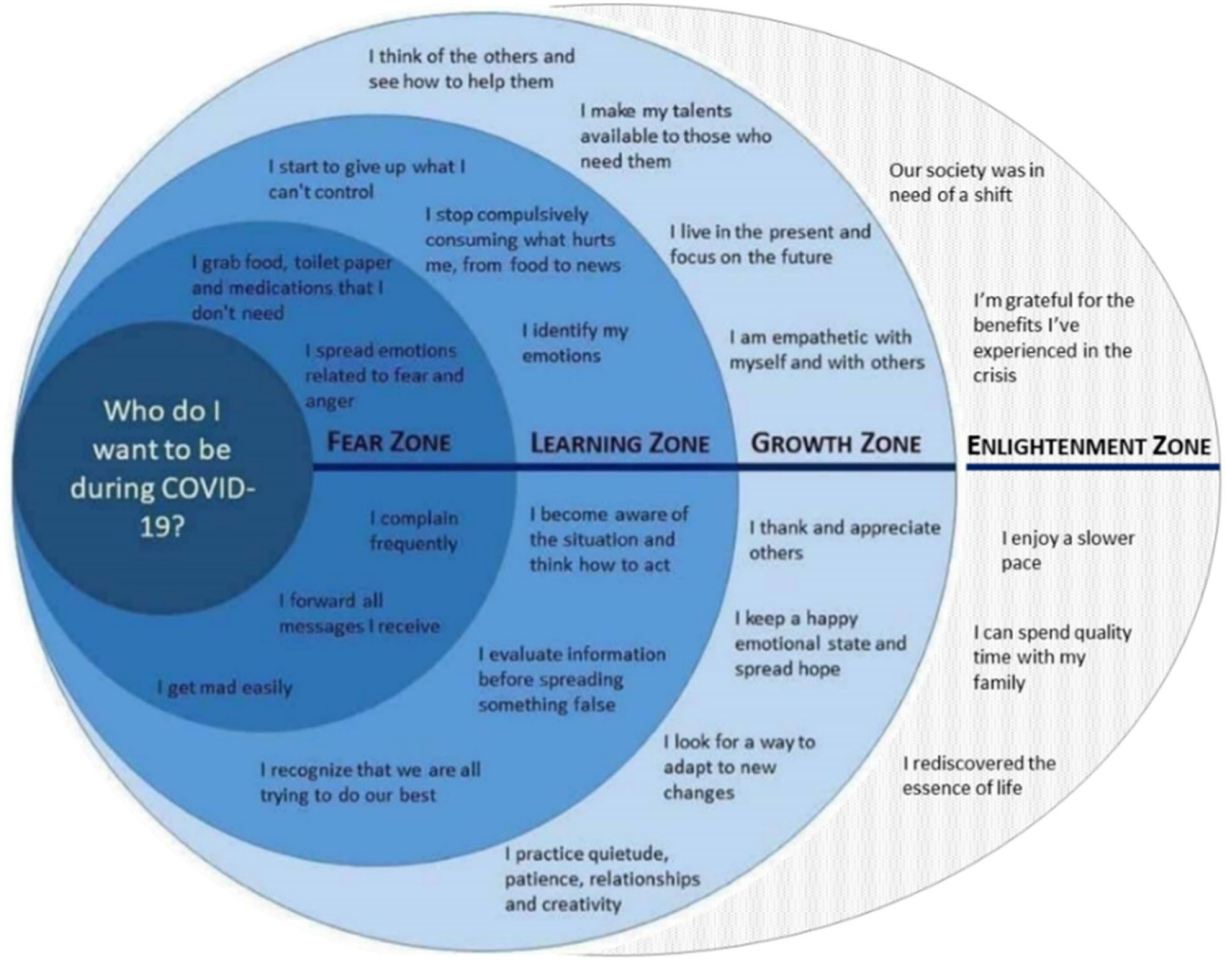
Conceptual zones in the metaphorical reframing process. (source: https://knowledge.insead.edu/blog/insead-blog/from-fear-to-enlightenment-building-resilience-during-covid-year-one-16346).

It suggests a demarcation line, where one needs to find a new direction in life. The reframing results in a novel conceptualization, giving a new perspective and new solutions for answers. In this metaphor we do not need commanders, weapons, isolation, in fact we need the opposite: collaboration, mutual support and respect, as it does not suggest WAR and a need for annihilation, but rather, a preventive, long-term view on life: it reminds us of sharing our planet with other organisms. Figure 11 shows the conceptual levels and zones of the metaphorization and the reframing process manifested in the above outlines research on different intra-cultural conceptualizations of the Covid metaphor through discourse contexts.

## Conclusions

We focused on Covid-19 conceptualizations and investigated universal and culture-specific features of conceptual metaphor in understanding and fighting it. We clarified the penetrating features of conceptual metaphors in human thinking (Lakoff and Johnson, 1980) and concluded that conceptual metaphors, being cognitive models, give us frames of conceptualization, which not only influences our concepts and beliefs but also have an inherent ability to change these. In other words, using a different metaphor to understand an abstract concept (e.g. pandemic) can trigger different conceptualizations, that influence cultures of local communities and eventually of societies at large and can therefore change our conceptualization to one that offers new perspectives, therefore, can help overcome the problem and *bring about social change.*

### General conclusions—Reframed conceptualization of Covid-19 metaphors in discourse context

**Fig. 12:**
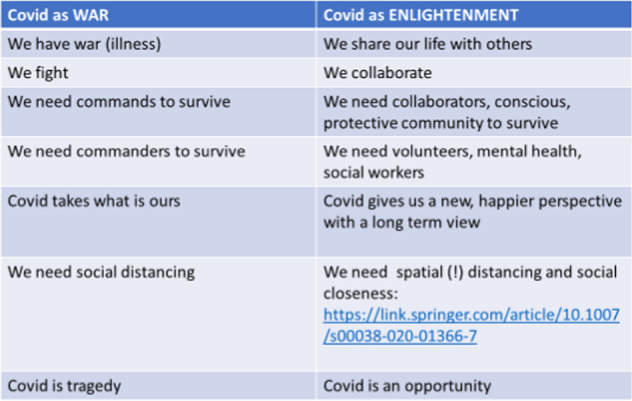
General summary and contrast of the two most prevalent Covid metaphors (COVID AS WAR VS. COVID AS ENLIGHTENMENT) conveyed in mass media discourse.

The conceptual metaphor we offer for Covid-19, contrary to the widespread COVID AS WAR metaphor in political discourse, is COVID AS ENLIGHTENMENT, which offers new solutions, perspectives and better chances of handling this social problem. Figure 12 shows the summary of the confronted two conceptualizations and their corresponding relevant features: COVID AS WAR vs. COVID AS ENLIGHTENMENT.

To conclude, our research indicates that these conceptual metaphors constitute powerful tools for social change in academic discourse, as they convey sharply different messages to people by reinforcing that Covid-19 is a tragedy vs. Covid-19 is an opportunity. This latter view calls for sharing our lives with each other, rather than social isolation. It calls for spatial distancing, rather than social distancing, and in fact reinforces the need to collaboration and social contact.

The list of websites used for the analysis of the conceptualizations of Covid19 are shown in Table 1.

**Table 1:** List of articles analyzed for the metaphorical conceptualizations of Covid-19.

1 https://www.nytimes.com/2020/04/20/opinion/coronavirus-war-politicians.html
2 https://www.versobooks.com/blogs/4636-states-of-emergency-metaphors-of-virus-and-covid-19
3 https://www.journal-psychoanalysis.eu/coronavirus-and-philosophers/5/
4 https://www.noemamag.com/from-the-anthropocene-to-the-microbiocene/
5 https://www.bbc.com/future/article/20200617-what-if-all-viruses-disappeared
6 https://www.wired.com/story/metaphors-matter-in-pandemic-coronavirus/
7 vaccines: https://www.euronews.com/2021/04/21/covid-19-vaccinations-in-europe-which-countries-are-leading-the-way; https://euobserver.com/opinion/151187
8. https://www.tandfonline.com/doi/full/10.1080/10410236.2020.1844989

### Conclusions of the EDUC joint course: a powerful tool in academic discourse for social change

The online collaborative joint course platform makes the participants more *open-minded*, acquiring an openness for different interpretations in *discourse*, where cultural differences give ground to very different expectations and thus strategies in *deciphering intentions* of the speaker and therefore, intended meanings (Schnell, 2007, 2019; Schnell et al., 2019). In such a *multicultural* milieu *academic discourse* triggered by the different perspectives yielded novel perspectives on *societal concerns*, therefore we believe that the *novel EDUC framework and the online joint course methodology* we applied is a powerful tool in education to bring about social change. Academic discourse is essential in this process as education is one of the most powerful platforms to *implement behavioral changes in societal concerns* and tackling challenges facing our century. Some sources on the EDUC Alliance and the joint course online methodology in academic discourse are in Table 2.

**Table 2:** EDUC sources on the online joint course.

• EDUC Alliance: https://educalliance.eu/
• University of Cagliari, Italy website:
• https://unica.it/unica/page/it/educ_partiti_i_primi_corsi_di_didattica_internazionale?fbclid=IwAR1agCWdbgH4zcCMDcyTCOOC_HwqYBFygNqsFueycZqnV5bhDVPnl-55ixk
• University of Pécs, Hungary website:
• https://kpvk.pte.hu/en/news/international_online_courses_university_cagliari_italy
• Reflections of students from the University of Pécs Faculty website:
• https://kpvk.pte.hu/en/news/reflections_students_educ_course
• Reflections of students from the University of Cagliari website:
• https://unica.it/unica/page/it/conclusi_i_primi_corsi_di_didattica_internazionale
• More news in the Italian Press on the academic collaboration, joint course methodology for social change and dissemination of knowledge across cultures in and outside the Academia (in Italian):
• https://webapi.unionesarda.it/articoloamp/cultura/2021/03/11/l-ateneo-di-cagliari-da-il-via-ai-primi-corsi-di-didattica-intern-8-1125132.html?fbclid=IwAR0lUdvS0tdA0aY-qcSwRt90PJhZxXuB4qfxu8JdNiNYqa1URrNUHnaB-b8
• #ReframeCovid Initiative, open-source document for Covid-19 reframing in discourse: https://sites.google.com/view/reframecovid/initiative

## Data Availability

The datasets generated during and/or analyzed during the current study are available from the corresponding author on reasonable request
